# Uptake of Cervical Cancer Screening Services and Its Association with Cervical Cancer Awareness and Knowledge Among Women of Reproductive Age in Dodoma, Tanzania: A Cross-Sectional Study

**DOI:** 10.24248/EAHRJ-D-19-00006

**Published:** 2019-11-29

**Authors:** Fabiola V Moshi, Musa Bago, Julius Ntwenya, Bonaventura Mpondo, Stephen M Kibusi

**Affiliations:** a University of Dodoma College of Health Sciences, Dodoma, Tanzania

## Abstract

**Background:**

There is a close link between an individual's knowledge about a given disease and uptake of screening and ultimately treatment. This study aimed to determine the link between knowledge levels and awareness and uptake of cervical cancer screening among women of reproductive age (15 to 49 years) in Dodoma, Tanzania.

**Methods:**

A cross-sectional study of 1,587 women aged between 15 and 49 years was conducted in Dodoma City, Tanzania. A structured questionnaire, adapted from Montgomery and others, was pretested and used to collect data from March to April, 2016 via multistage sampling. Univariate and multiple regression analyses were used to determine factors associated with the level of knowledge about cervical cancer and the association between knowledge and uptake of cervical cancer screening.

**Results:**

The mean age of the participants was 26.99±8.026 years. Only 165 (10.4%) of the 1,587 participants were knowledgeable about cervical cancer; 1,051 (66.2%) were aware of cervical cancer screening, and only 125 (7.9%) had undergone cervical cancer screening. Predictors of knowledge about cervical cancer were education level (secondary education adjusted odds ratio [AOR] 2.23; 95% confidence interval [CI], 1.030–4.811; *P*<.05; university level AOR 2.59; 95% CI, 1.179 to 5.669; *P*<.05); residence (rural AOR 1.85; 95% CI, 1.282 to 2.679; *P*=.001); parity (multipara AOR 1.88; 95% CI, 1.125 to 3.142; *P*<.05).

After adjusting for confounders, knowledge about cervical cancer significantly influenced both cervical cancer screening awareness (AOR 2.91; 95% CI, 1.821 to 4.640; *P*<.001) and uptake (AOR 2.065; 95% CI, 1.238 to 3.444; *P*=.005).

**Conclusion:**

The level of knowledge about cervical cancer was extremely low. Women with less knowledge about cervical cancer were those with less education, those living in rural areas, and those without children. A low level of knowledge was associated with poor uptake of screening services, highlighting the need for integrating health education pertaining to cervical cancer and screening when providing reproductive health care in Tanzania.

## INTRODUCTION

Cervical cancer is well recognised as the third most common cancer among women. In 2012, it accounted for 265,700 deaths worldwide.^1^ More than 85% of the documented deaths were reported in developing countries, and up to 60,100 deaths occurred in Africa.^1^ Cervical cancer contributes up to 9% of all new cases of cancer in women worldwide.^2^ In Africa, the East African Region in particular has the highest incidence rates of cervical cancer, with 42.7 new cases per 100,000 women.^1^ In 2016, Tanzania reported an incidence rate of 50.9/100,000, which was the highest rate in the East African Region.^4^ The high cervical cancer incidence in Tanzania can be attributed to high prevalence of human papillomavirus (HPV) infection and low rates of disease screening.^5^

Nearly all cases of cervical cancer can be attributable to HPV infection.^3^ Most cervical cancers are caused by persistent infection with HPV types 16, 18, and 45, which are the most common types of HPV and cause 75% of all squamous cell carcinoma and 94% of all adenocarcinoma.^6^ Early sexual debut or multiple sexual partners and unprotected sex are the most cited risk factors for HPV infection and cervical cancer.^7^ Both persistence of HPV infection and the progression to cancer may be influenced by many factors, including immunosuppression, high parity, cigarette smoking, long-term use of oral contraceptives, intrauterine device use, and being younger than 17 years at full-term pregnancy.^7^

Despite its epidemiological importance, cervical cancer in Tanzania has not received the public health attention it deserves. The national health policy in Tanzania does not have a screening policy for cervical cancer; rather, priority is given to infectious diseases, such as malaria, tuberculosis, leprosy, diarrhoeal diseases, acute respiratory infections, and sexually transmitted infections—all of which have individual control programmes.^8^ However, recently, there have been public awareness campaigns through public announcements of the HPV preventive measures and screening programme. The national strategic plan for 2015–2020 stipulated that by 2020, 80% of women aged 30 to 50 years will be screened for cervical cancer.^9^ The Tanzanian Ministry of Health plans to increase attention to public awareness campaigns and increase the demand for screening and early detection and treatment of cervical cancer.^9^ By 2020 all council hospitals will offer cervical cancer screening.^9^ Determining the groups of women who remain unfamiliar with cervical cancer is crucial for educational messages to benefit those in most need.^10^

Poor cervical cancer knowledge has widely been reported as a risk factor for high cervical cancer incidence and mortality.^11^ Researchers have found that women with high screening rates have increased chances of early diagnosis and early treatment.^10^

A woman's level of education,^13,14^ perception regarding her vulnerability to HPV infection,^15^ low socioeconomic status,^13,16^ residential setting (rural or urban),^16,17^ and young age^14,18^ are factors associated with knowledge about HPV infection and cervical cancer. Knowledge about cervical cancer,^19,20^ income levels,^21^ fear of Pap smear,^22^ age,^19,23^ place of residence,^23^ and distance to the nearest facility providing cervical cancer screening^20^ are factors that influence cervical cancer screening uptake. Cervical cancer screening is associated with a reduced incidence of invasive cervical cancer and deaths due to cervical cancer.^24^ There is a scarcity of literature reporting knowledge levels, screening awareness, and uptake of cervical cancer screening in Tanzania. Therefore, this study aimed to determine knowledge levels, screening awareness, and uptake rates of cervical cancer screening among women of reproductive age (15-49 years) in Dodoma City, Tanzania.

## METHODS

### Study Design and Setting

A descriptive cross-sectional study was conducted between March and April 2016 in Dodoma City, Tanzania. The City is subdivided into 4 divisions (Urban, Hombolo, Kikombo and Zuzu Division) comprising of a total of 30 wards and 42 villages/streets. Dodoma Region lies in the eastern-central part of Tanzania and it is the capital city in the country.

Dodoma is a semi-arid climate region with relatively warm temperatures throughout the year. The average temperature is 22.6°C whereby the lowest dips to 13°C. The precipitation averages 570mm per year, the bulk of which occurs during wet season between November and April whereby the remainder of the year comprises the city's dry season.

According to the 2012 national census; the City has a population of 199,487 males and 211,469 females making a total of 410,956 people.

The City has 15 health facilities which fall under secondary and tertiary health-care system and they hold the capacity for cervical cancer screening but unfortunately only 2 hospitals out of the 15 are providing the services (Dodoma Regional Hospital and Makole Health Centre).

### Target Population

Women of reproductive age (15 years – 49 years) in Dodoma City

### Study Population

Women of reproductive age (15-49 years) residing in Dodoma City during the time of study were eligible to participate. We excluded women who were seriously ill and not able to answer questions.

### Sample Size and Sampling Technique

The sample size was calculated using Fischer's/Lesley formula, n=z^2^p(1−p)/e^2^ where, n is sample size, z= standard normal deviation set at 1.96 at 95% confidence interval (CI), p= proportion of knowledge in previous study and e= maximum error, assumed to be 5%

p= 69.1% prevalence of knowledge about cervical cancer in Tanzania^26^

Hence, $\begin{array}{l} \text{n}=\frac{{\left(1.96\right)}^{2}*0.69(1-0.69)}{{\left(0.05\right)}^{2}}\\ \text{n}=328 \end{array}$

Attrition rate of 25% was added to the calculated sample size to have the sufficient number of respondents, therefore; in each ward

25% of 328 is 82

328+ 82=410

The minimum sample size was 394 women from each city division. The city has 4 divisions. Therefore, the sample size was 410*4 =1,640

A multistage sampling was used to select sample units from the population, starting with randomly selection of 20 wards out of 30 wards; 5 wards from each division. The second stage sampling were selection of streets, where 4 streets from each selected ward were selected. From a total of 56 streets, in each street 31 to 32 households were taken by lottery method and if more than 1 eligible woman was present in the same household, 1 was recruited using lottery method using a list of residents provided by Ward local authorities. This number was sufficient to give the needed sample size of 1,576 study participants.

### Data Collection Method

A quantitative method was employed whereby data was collected using a self-administered structured questionnaire. The questionnaire had 4 parts. The first part was about socio-demographic characteristics, the second part was knowledge questions, the third part was awareness about screening question and the last part was the uptake question. Knowledge about cervical cancer was assessed using 20 questions covering the meaning of cervical cancer, who are at risk, signs and symptoms, pre disposing factors, modes of transition, how can it have prevented, when the cervical cancer is treatable. The awareness about screening was assessed by only 1 question which asked the respondents on whether they had ever heard about cervical cancer. The uptake was assessed on whether they had ever uptake the screening.

The questionnaire was developed in English and translated into a Swahili version which was administered to the participants. The questionnaire was pretested on 20 participants to ensure validity and reliability. Questionnaire was administered by the interviewer to a participant who spent nearly 30 minutes to respond and return back the filled questionnaire to the interviewer soon after completion.

### Data Analysis Procedure

Data were checked first for completeness and missing information and then manually cleaned before they were analysed using SPSS, Version 20 (IBM Corp., Armonk, NY, USA). Demographic characteristics were analysed by descriptive statistics to indicate frequencies and percentage. Chi-squire analysis was used to test the relationship between sociodemographic characteristics and cervical cancer knowledge and the relationship between cervical cancer knowledge, screening awareness, and uptake of screening. Knowledge about cervical cancer was assessed using a criterion that those who scored 50% of the total points (10points) and above had adequate knowledgeable and those who scored below 50% (less than 10points) had inadequate knowledge. On awareness of screening was assessed using only 1 question on whether had ever heard about cervical cancer screening. Those who responded yes were taken as being aware and those who responded no were taken as not aware. On assessing the uptake was assessed using 1 question on whether had ever screened on not. Multivariate analysis was used to control the confounders. Adjusted odds ratios (AORs) were used to determine the strength of association between the selected variables. Significance level was 0.05 (*P*<.05), and the confidence interval of 95% was used to determine the significance of association between knowledge and screening awareness and uptake.

### Ethical Considerations

The ethical clearance approval was given by the University of Dodoma, Institutional Research Review Committee with reference number UDOM/DRP/IRRC/14/VOL V/20 offered on 10^th^ February 2016. The written informed consent was obtained from each respondent. The oral informed assent was sought from the parents or guardians of participants who were younger than 18years. To ensure confidentiality and anonymity, neither the names of respondents nor those of institutions involved were requested on the questionnaire.

## RESULTS

### Sociodemographic Characteristics

Overall 1,587 women responded from a total of 1640 questionnaires distributed, giving a response rate of 96.8%. The maximum age was 49 years while the minimum age was 15 years with a mean age of 26.99 (SD 8.026). The majority of the respondents 554 (34.9%) aged 20-24. Almost half 667 (42%) of the respondents were married. In terms of education level, 536 (33.8%) of them received primary education. Most of respondents (n=1,158, 73%) were unemployed and 1,026 (64.7%) of them had no monthly income. Most of the respondents (n=1,125, 70.9%) were Christians while the majority of them (n=1,309, 82.5%) reside in urban setting. The majority of the respondents (n=1,308, 82.4%) reported some health-seeking behaviour, and the majority (n=1,250, 78.8%) of them had never seen anyone suffering from cervical cancer (Table 1).

**TABLE 1.** Distribution of Study Participants by Sociodemographic Characteristics (N=1,587)

Variable	n	%
**Age group**		
15–19	241	15.2
20–24	554	34.9
25–29	299	18.8
30–34	212	13.4
35–39	118	7.4
40–44	93	5.9
45–49	70	4.4
**Marital status**		
Not married	550	34.7
Married	667	42.0
Divorced	114	7.2
Co-habiting	256	16.1
**Education level**		
None	175	11.0
Primary	536	33.8
Secondary	353	22.2
University	523	33.0
**Occupation**		
None	222	14.0
Not employed	1158	73.0
Others	207	13.0
**Monthly income**		
Less than 100 USD	374	23.6
More than 100 USD	187	11.8
No income	1026	64.7
**Religion**		
Christian	1125	70.9
Muslim	460	29.0
Pagan	2	0.1
**Residence**		
Urban	1309	82.5
Rural	278	17.5
**Health-seeking behaviour**		
Yes	1308	82.4
No	279	17.6
**Seeking for any test**		
Always	413	26.0
Rarely	992	62.5
Not seeking	182	11.5
**Seen anyone with cervical cancer**		
Yes	337	21.2
No	1250	78.8

### The Relationship Between Sociodemographic Characteristics and Cervical Cancer Knowledge

Out of 1,587 study participants, 165 (10.4%) of them had some knowledge about cervical cancer. Cervical cancer knowledge was significantly associated with age (*P*=.007), level of education (*P*=.03), occupation status (*P*=.02), income (*P*<.001), health-seeking behaviour (*P*<.001), residence (*P*=.01) and if one has ever seen anyone with cervical cancer (*P*<.001). The marital status and religion of the participants were not statistically significant (Table 2).

**TABLE 2.** Relationship Between Sociodemographic Characteristics and Knowledge (N=1,587)

Variables	Knowledge		χ^2^	*P* Value
	High n (%)	Low n (%)		
**Age group**				
15–19	18 (7.5)	223 (92.5)	12.364	.007
20–24	50 (9)	504 (91)		
25–29	33 (11)	266 (89)		
30–34	24 (11.3)	188 (88.7)		
35–39	15 (12.7)	103 (87.3)		
40–44	18 (19.4)	75 (80.6)		
45–49	7 (10)	63 (90)		
**Marital status**				
Unmarried	52 (9.5)	498 (90.5)	0.878	.42
Married	72 (10.8)	595 (89.2)		
Divorced	12 (10.5)	102 (89.5)		
Cohabiting	29 (11.3)	227 (88.7)		
**Education level**				
None	11 (6.3)	164 (93.7)	8.903	.03
Primary	46 (8.6)	490 (91.4)		
Secondary	41 (11.6)	312 (88.4)		
University	67 (12.8)	456 (87.2)		
**Occupation**				
Employed	45 (20.3)	177 (79.7)	29.066	.02
None	96 (8.3)	1,062 (91.7)		
Other	24 (11.6)	183 (88.4)		
**Monthly income**				
<100USD	39 (10.4)	335 (89.6)	18.646	<.001
>100USD	36 (19.3)	151 (80.7)		
None	90 (8.8)	936 (91.2)		
**Religion**				
Christian	115 (10.2)	1010 (89.8)	0.379	.746
Muslim	50 (10.9)	410 (89.1)		
Others	0 (0.0)	2 (100)		
**Health-seeking behaviour**				
Yes	153 (11.7)	1155 (88.3)	13.503	<.001
No	12 (4.3)	267 (95.7)		
**Residence**				
Urban	151 (11.5)	1158 (88.5)	10.398	.01
Rural	14 (5)	264 (95)		
**Seen anyone with cervical cancer**				
Yes	58 (17.2)	279 (82.8)	21.322	<.001
No	107 (8.6)	1143 (91.4)		

### Predictors of Cervical Cancer Knowledge

After controlling the confounders in logistic regression, participants with higher levels of formal education (secondary level, AOR 2.23; 95%CI, 1.030 to 4.811; *P*=.042, university education level, AOR 2.59; 95%CI, 1.179 to 5.669, *P*=.018) were more likely to be knowledgeable about cervical cancer compared with women who had no formal education. The second predictor was the place of residence (urban, AOR 2.26; 95%CI, 1.265 to 4.029; *P*=.006). Having seen anyone with cervical cancer was the third predictor of knowledge as women who had ever seen anyone with cervical cancer (AOR 1.85; 95%CI, 1.282 to 2.679; *P*=.001) were more likely to be knowledgeable about cervical cancer compared with women who had never seen anyone with cervical cancer. The last predictor was parity as women who had more than 1 child (AOR 1.88; 95%CI, 1.125 to 3.142; *P*=.016) were more likely to be knowledgeable about cervical cancer compared with women with no children (Table 3).

**TABLE 3.** Predictors of Cervical Cancer Knowledge Among Women of Reproductive Age in Dodoma, Tanzania (N=1,587)

Variable	Adjusted Odds Ratio	CI	*P* Value
**Age group**			
15–19	1		
20–24	1.03	0.565–1.885	.917
25–29	1.25	0.633–2.461	.523
30–34	1.22	0.568–2.622	.610
35–39	1.38	0.576–3.307	.470
40–44	2.23	0.927–5.348	.073
45–49	1.15	0.403–3.288	.793
**Marital status**			
Not Married	1		
Married	0.73	0.433–1.227	.235
Divorced	0.87	0.397–1.913	.732
Cohabited	1.10	0.668–1.807	.710
**Education level**			
None	1		
Primary Level	1.36	0.668–2.786	.395
Secondary Level	2.23	1.030–4.811	.042
University Level	2.59	1.179–5.669	.018
**Occupation**			
Others	1		
Employed	1.15	0.596–2.214	.679
None	0.57	0.327–1.009	.054
**Monthly income**			
No Income	1		
Less than 100 USD	0.76	0.443–1.312	.327
More than 100 USD	0.87	0.421–1.776	.692
**Residence**			
Rural	1		
Urban	2.26	1.265–4.029	.006
**Seen Anyone with cervical cancer**			
No	1		
Yes	1.85	1.282–2.679	.001
**Parity**			
Nulliparous	1		
Primiparous	0.99	0.587–1.695	.992
Multiparous	1.88	1.125–3.142	.016

### Association Between Cervical Cancer Knowledge and Cervical Cancer Screening Awareness

The majority of study participants (n=1,051, 66.2%) were aware about cervical cancer screening. A chi-square test results showed a significant relationship between knowledge about cervical cancer and screening awareness (*P*<.001). Other factors including, age groups (*P*<.001), education level (*P*<.001), occupation (*P*<.001), monthly income (*P*<.001), residence (*P*=.002), having seen anyone with cervical cancer (*P*<.001) showed significant relationship with screening awareness. The marital status, religion of participant and parity were not statistically significant (see table 4).

**TABLE 4.** Relationship Between Sociodemographic Characteristics and Screening Awareness (N=1,587)

Variables	Aware n (%)	Not Aware n (%)	χ^2^	*P* Value
**Age group**				
15–19	144 (59.8)	97 (40.2)	30.863	<.001
20–24	356 (64.3)	198 (35.7)		
25–29	218 (72.9)	81 (27.1)		
30–34	149 (70.3)	63 (29.7)		
35–39	73 (61.9)	45 (38.1)		
40–44	75 (80.6)	18 (19.4)		
45–49	35 (50)	35 (50)		
**Marital status**				
Not Married	349 (63.5)	201 (36.5)	4.753	.191
Married	445 (66.7)	222 (33.3)		
Divorced	74 (64.9)	40 (35.1)		
Cohabiting	182 (71.1)	74 (28.9)		
**Education level**				
None	95 (54.3)	80 (45.7)	21.897	<.001
Primary	343 (64)	193 (36)		
Secondary	261 (73.9)	92 (26.1)		
University	351 (67.1)	172 (32.9)		
**Occupation**				
Employed	176 (79.3)	46 (20.7)	19.989	<.001
None	739 (63.8)	419 (36.2)		
Others	135 (65.2)	72 (34.8)		
**Monthly income**				
<100USD	268 (71.7)	106 (28.3)	24.883	<.001
>100USD	146 (78.1)	41 (21.9)		
No Income	636 (62)	390 (38)		
**Religion**				
Christian	731 (65)	394 (35)	2.749	.253
Muslim	318 (69.1)	142 (30.9)		
Other	1 (50)	1 (50)		
**Residence**				
Urban	888 (67.8)	421 (32.2)	9.370	.002
Rural	162 (58.3)	116 (41.7)		
**Seen anyone with cervical cancer**				
Yes	263 (78)	74 (22)	26.968	<.001
No	787 (63)	463 (37)		
**Parity**				
Nulliparous	455 (66.5)	229 (33.5)	0.085	.958
Primiparous	217 (66.2)	111 (33.8)		
Multiparous	378 (65.7)	197 (34.5)		
**Level of knowledge about cervical cancer**				
High	142 (86.1)	23 (13.9)	32.567	<.001
Low	908 (63.9)	514 (36.1)		

After controlling the confounders (sociodemographic characteristics) in logistic regression, there was a significant relationship between level of knowledge (AOR 2.91; 95% CI, 1.821 to 4.640; *P*<.001) and awareness of cervical cancer screening (Table 5).

**TABLE 5.** Association Between Cervical Cancer Knowledge and Cervical Cancer Screening Awareness (N=1,587)

Variable	Adjusted Odds Ratio	CI	*P* Value
**Age group**			
15–19	1		
20–24	1.27	0.900–1.798	.172
25–29	1.89	1.244–2.871	.003
30–34	1.89	1.171–3.034	.009
35–39	1.24	0.708–2.156	.456
40–44	2.68	1.392–5.175	.003
45–49	0.88	0.468–1.649	.688
**Marital status**			
Not Married	1		
Married	1.04	0.742–1.452	.829
Divorced	1.16	0.690–1.934	.584
Cohabited	1.40	0.997–1.952	.052
**Education level**			
No Education	1		
Primary	1.30	0.896–1.893	.167
Secondary	2.15	1.390–3.336	.001
University	1.40	0.897–2.155	.141
**Occupation**			
Others	1		
Employed	1.21	0.717–2.023	.482
Not Employed	1.18	0.810–1.724	.387
**Monthly income**			
No Income	1		
<100USD	1.47	1.041–2.077	.029
>100USD	1.61	0.958–2.714	.72
**Residence**			
Rural	1		
Urban	1.36	1.024–1.801	.033
**Seen anyone with cervical cancer**			
No	1		
Yes	1.872	1.391–2.521	<.001
**Level of knowledge**			
Low	1		
High	2.91	1.821–4.640	<.001
**Parity**			
Nulliparous	1		
Primiparous	0.76	0.549–1.050	.095
Multiparous	0.76	0.539–1.064	.109

### Associations With Screening Uptake

Out of 1,587 of the study participants, only 125 (7.9%) had uptake cervical cancer screening. The results of univariate analysis by chi-square test showed that age group (*P*=.02), marital status of participant (*P*<.001), educational level (*P*=.018), occupation status (*P*<.001), monthly income (*P*<.001), residence (*P*=.007), having seen anyone with cervical cancer (*P*<.001), and level of knowledge about cervical cancer (*P*<.001) were significantly related to uptake of cervical cancer screening (Table 6).

**TABLE 6.** Relationship Between Sociodemographic Characteristics and Uptake of Cervical Cancer Screening (N=1,587)

Variables	Ever Screened n (%)	Never n (%)Screened	χ^2^	*P* Value
**Age group**				
15–19	9 (3.7)	232 (96.3)	20.447	.002
20–24	35 (6.3)	519 (93.7)		
25–29	34 (11.4)	265 (88.6)		
30–34	18 (8.5)	194 (91.5)		
35–39	12 (10.2)	106 (89.8)		
40–44	14 (15.1)	79 (84.9)		
45–49	4 (5.7)	66 (70)		
**Marital Status**				
Unmarried	26 (4.7)	524 (95.3)	34.794	<.001
Married	84 (12.6)	583 (87.4)		
Divorced	3 (2.6)	111 (97.4)		
Cohabiting	13 (5.1)	243 (94.9)		
**Education level**				
None	6 (3.4)	169 (96.6)	10.128	.018
Primary	44 (8.2)	492 (91.8)		
Secondary	39 (11)	314 (89)		
University	37 (7.1)	485 (92.9)		
**Occupation**				
Employed	44 (19.8)	178 (80.2)	50.032	<.001
Unmployed	68 (5.9)	1,090 (94.1)		
Others	14 (6.8)	193 (93.2)		
**Monthly income**				
<100USD	45 (12)	329 (88)	40.858	<.001
>100USD	31 (16.6)	156 (83.4)		
No Income	50 (4.9)	976 (95.1)		
**Religion**				
Christian	85 (7.6)	1,040 (92.4)	0.996	.608
Muslim	41 (8.9)	419 (91.1)		
Others	0 (0.0)	2 (100)		
**Residence**				
Urban	114 (8.7)	1,195 (91.3)	6.053	.007
Rural	12 (4.3)	266 (95.7)		
**Health-seeking behaviour**				
Yes	108 (8.3)	1,200 (91.7)	1.025	.188
No	18 (6.5)	261 (93.5)		
**Seen anyone with cervical cancer**				
Yes	69 (20.5)	268 (79.5)	91.981	<.001
No	57 (4.6)	1193 (95.4)		
**Parity**				
Nulliparous	28 (4.1)	656 (95.9)	29.168	<.001
Primiparous	27 (8.2)	301 (91.8)		
Multiparous	71 (12.3)	504 (87.7)		
**Level of knowledge about cervical cancer**				
High	29 (17.6)	136 (82.4)	23.394	<.001
Low	97 (6.8)	1325(93.2)		

### Association Between Cervical Cancer Screening Knowledge and Uptake of Screening

After controlling for confounders (sociodemographic characteristics), there was a significant association between knowledge about cervical cancer (AOR 2.065; 95% CI, 1.238 to 3.444; *P*=.005) and uptake of cervical cancer screening (Table 7).

**TABLE 7.** Association Between Cervical Cancer Knowledge and Cervical Cancer Screening Uptake (N=1,587)

Variable	Adjusted Odds Ratio	CI	*P* Value
**Age group**			
15–19	1		
20–24	1.53	0.681–3.447	.302
25–29	1.44	0.600–3.442	.415
30–34	0.74	0.277–1.955	.539
35–39	0.86	0.291–2.526	.779
40–44	0.76	0.256–2.232	.613
45–49	0.52	0.132–2.039	.347
**Marital status**			
Not Married	1		
Married	1.64	0.885–3.018	.116
Divorced	0.30	0.80–1.134	.076
Cohabited	0.91	0.446–1.886	.814
**Education level**			
No Education	1		
Primary	1.91	0.772–4.803	.160
Secondary	2.51	0.952–6.627	.063
University	1.55	0.554–4.317	.405
**Occupation**			
Others	1		
Employed	1.90	0.871–4.159	.107
Not Employed	1.21	0.610–2.414	.581
**Monthly income**			
No Income	1		
<100USD	1.73	0.996–3.005	.052
>100USD	1.66	0.769–3.594	.197
**Residence**			
Rural	1		
Urban	1.62	0.845–3.594	.146
**Seen anyone with cervical cancer**			
No	1		
Yes	4.584	3.033–6.926	.000
**Level of Knowledge**			
Low	1		
High	2.065	1.238–3.444	.005
**Parity**			
Nulliparous	1		
Primiparous	1.32	0.698–2.488	.395
Multiparous	2.39	1.300–4.391	.005

## DISCUSSION

This study aimed at assessing the level of knowledge, screening awareness and uptake of cervical cancer.

The study revealed the low level of knowledge about cervical cancer among women of reproductive age. Among 1,587 women who were interviewed only 10.4% of them were knowledgeable about cervical cancer. Similar study done in Nigeria reported even lower proportion of women who were knowledgeable about cervical cancer; of 240 women interviewed, only 4.2% were knowledgeable about cervical cancer.^15^ A different finding was reported in another study done in Ethiopia, where 31% of study participants had sufficient knowledge about cervical cancer.^12^ The possible reasons for the difference in the findings could be due to different study locations, the tools used for data collection and different sample sizes. In another study also done in Ethiopia reported that majority of study participants had ever heard about cervical cancer but only 49% knew the causes.^26^ The difference in the finding could be due to different study populations whereby the current study interviewed all women in reproductive age while the study in Ethiopia interviewed only Human Immunodeficiency Virus (HIV) positive women.

This study also revealed several factors which influence the level of knowledge about cervical cancer. Level of education, place of residence, seen anyone with cervical cancer and parity of a woman were predictors of level of knowledge a woman had about cervical cancer. Women who had more than primary education were more likely to be aware about cervical cancer compared to women who had no formal education, similar findings were reported by Mitiku et al,^14^ Getahun et al.^13^ This emphasises the importance of education in generating awareness about cervical cancer among the population at large.

Another factor which influenced the level of knowledge about cervical cancer was place of residence. Women who reside in urban area were more than twice likely to be knowledgeable about cervical cancer compared with women who lives in rural area. This finding correspond with finding from another study done in Kinshasa.^27^ This could be explained that women who reside in urban have more access to health information through media and ease access to health care as compared to those who are living in rural areas.

Accordingly, this study having had a personal, familiar, or friendly history of cervical cancer were significant associated with an increased knowledge about cervical cancer, this is similar to findings from another study.^19^ A similar study in Ethiopia reported that, women who knew someone affected with cervical cancer were about 3.4 times more likely to have above median knowledge than who did not.^14^ This indicates that knowing person/women with the case helps the other people/women to know more about the diseases by reading or getting the information/education from those women who are affected by the disease.

A different finding was reported in a study done in Ethiopia reported that age, marital status, religion, experienced sexual intercourse and age at 1st sexual intercourse were found to be significantly associated with the knowledge of cancer of the cervix.^29^

The odds of respondents in the age range of 35–49 years being knowledgeable about cervical cancer were 2.8 times more as compared to other age groups. Protestant religion followers were 8.8 times more likely to be knowledgeable than other religion followers. Those who had no history of sexual intercourse were almost 3 times more likely to be knowledgeable than those who had sexual intercourse and from those who had experienced sexual intercourse at the age ≥18 years were 3.7 more likely to be knowledgeable than their counterparts.^29^

The study also found that majority of study participants 66.2% were aware of cervical cancer screening. The level of knowledge about cervical cancer was an important determinant for the awareness of cervical cancer screening. A similar study done in India reported that among those who were aware of cervical cancer, only 47(69.1%) knew about the existence of a screening test to detect cancer cervix. But when knowledge, attitude, and practice regarding Pap smear were assessed among those who had heard about the test. More than half (57.45%) did not know what kind of test it was. Among those claiming knowledge about Pap smear, 23.4% thought it was a blood test, 17.02% a type of biopsy and 2% a urine test.^30^

A different finding was reported in a study done in Bangladesh revealed only 16.1% respondents reported that the cervical cancer can be detected by screening. The study also found that women were very poorly aware of VIA and Pap smear test as screening methods; only 10.2% and 7.6% respectively.^31^ Another study done in Finote selam, Ethiopia reported that from all study participants, only 142 (19.3%) had heard about screening for cervical cancer and from these 54 (38%) were screened for the disease.^29^ The probable discrepancy here maybe, because of the study setting difference where Dodoma City is an urban setting whereas Finote selam is relatively a rural town.

This revealed that very few women of reproductive age had an uptake of cervical cancer screening (7.9%). This is a worrisome finding because Tanzania is leading in East Africa with the highest incidence of cervical cancer which is highly contributed by high prevalence of HPV virus.^5^ A similar study done in Kalkata showed very low (1.7%) uptake of cervical cancer screening. Another study done in Tamil Nadu also reported that only 17.02% had ever done the Pap test, though 47% of the population knew that cervical cancer could be detected early by a screening test.^30^

The study also revealed that there is a significant relationship between knowledge about cervical cancer and uptake of screening. This shows that many women are not taking the screening because of lack of information about cervical cancer. An innovative strategy has to be employed to raise awareness about cervical cancer to serve these women from a treatable cancer. A number of studies from other sub-Saharan African countries have similarly found that women who had low cervical cancer-related knowledge are less likely to be aware and participate in screening services.^21,28^ But also another study done in Zimbabwe revealed a significant positive correlation (r=.187; *P*<.001) between knowledge of cervical cancer and uptake of screening. The results mean that uptake of cervical cancer screening in this particular study increased as knowledge of cervical cancer increased.^33^

Despite the fact that the awareness of cervical cancer was high, it was not a necessary factor for the uptake of cervical cancer screening. The lack knowledge about cervical cancer can be partly explained that, the disease has not received the attention it deserves despite the fact that it is the most common female cancer in sub-Saharan Africa.^20^

The study also found that seen anyone with cervical cancer influenced knowledge about cervical cancer, awareness and uptake of cervical cancer screening. Women who had ever seen anyone with cervical cancer 85% more likely to be knowledgeable about cervical cancer, 87% more likely to be aware of cervical cancer screening and more than 3 times to uptake of cervical cancer screening compared to women who had never seen anyone with cervical cancer. Similar finding by Ndejjo et al^30^ reported a strong relationship between seen anyone with cervical cancer and the uptake of cervical cancer screening.

### Limitations

Based on this study, it is difficult to establish cause-effect relationship since the exposure and outcome measures are collected simultaneously.

The study was conducted during day time when most of the working women were not available at home. Hence, they were under-represented in the sample. This also made it difficult to reach out the calculated sample of 1800 participants.

Also, time allocated for the data collection process was limited such that, was not enough for the huge sample that was needed for this study causing the discrepancy in the calculated sample and the actual sample that responded to the questionnaires.

## CONCLUSION

Despite of high awareness about cervical cancer screening, uptake of the screening among women was very low. Low knowledge was associated with poor uptake of the screening highlighting the need for integrating health education package pertaining cervical cancer and it's respective screening when providing reproductive health care in Tanzania. The health education should emphasis on the risk factors of the disease as it is the essential key to ensure the preventive measures to be taken. The importance of routine cervical cancer screening must also be highlighted.

There is urgent need to improve awareness as well as provide affordable cervical cancer screening services. Also it is important to raise awareness among population on HPV and its link to cervical cancer. Flyers and pamphlets on HPV infection and cervical cancer should be available in family planning clinics. But also the role of mass media campaign (use of FM radio which is currently very available) should be considered in creating public awareness to further the knowledge by educating about risk factors, sign and symptoms, prevention methods of the cancer and benefit of screening test.

Findings of this study might help to increase uptake of screening of cervical cancer and early treatment. The findings will help in identifying relevant approaches to reduce the gap between knowledge and practice. The study therefore will also provide information that can be used in national strategic planning for cervical cancer prevention, for example, direction of focus in cervical cancer prevention.

## References

[1] Torre LA, Bray F, Siegel RL, Ferlay J, Lortet-Tieulent J, Jemal A. Global cancer statistics, 2012. CA Cancer J Clin. 2015;65(2):87–108. 10.3322/caac.21262. Medline 25651787

[2] Jemal A, Bray F, Center MM, Ferlay J, Ward E, Forman D. Global cancer statistics. CA Cancer J Clin. 2011;61(2):69–90. 10.3322/caac.20107. Medline 21296855

[3] Greer BE. Cervical Cancer Screening and Treatment in Tanzania. Vol 1. New Windsor, NY, USA; 2016.

[4] Jemal A, Center MM, DeSantis C, Ward EM. Global patterns of cancer incidence and mortality rates and trends. Cancer Epidemiol Biomarkers Prev. 2010;19(8):1893–1907. 10.1158/1055-9965.EPI-10-0437. Medline 20647400

[5] Bray F, Ren JS, Masuyer E, Ferlay J. Global estimates of cancer prevalence for 27 sites in the adult population in 2008. Int J Cancer. 2013;132(5):1133–1145. 10.1002/ijc.27711. Medline 22752881

[6] de Sanjose S, Quint WG V, Alemany L, et al. Human papillomavirus genotype attribution in invasive cervical cancer: a retrospective cross-sectional worldwide study. Lancet Oncol. 2010;11(11):1048–1056. 10.1016/S1470-2045(10)70230-8. Medline 20952254

[7] American Cancer Society (ACS). What is cervical cancer? ACS Website. http://www.cancer.org/cancer/cervicalcancer/detailedguide/cervical-cancer-what-is-cervical-cancer.

[8] United Republic of Tanzania Ministry of Health (MoH). National Health Policy. Dar es Salaam, Tanzania: MoH; 2003. http://apps.who.int/medicinedocs/documents/s18419en/s18419en.pdf.

[9] United Republic of Tanzania Ministry of Health and Social Welfare. Health Sector Strategic Plan July 2015 – June 2020 (HSSP IV). Dar es Salaam, Tanzania; 2015. http://www.tzdpg.or.tz/fileadmin/documents/dpg_internal/dpg_working_groups_clusters/cluster_2/health/Key_Sector_Documents/Induction_Pack/Final_HSSP_IV_Vs1.0_260815.pdf.

[10] Rama CH, Villa LL, Pagliusi S, et al. Awareness and knowledge of HPV, cervical cancer, and vaccines in young women after first delivery in São Paulo, Brazil–a cross-sectional study. BMC Womens Health. 2010;10:35. 10.1186/1472-6874-10-35. Medline PMC3022825 21176230

[11] Getahun F, Mazengia F, Abuhay M, Birhanu Z. Comprehensive knowledge about cervical cancer is low among women in Northwest Ethiopia. BMC Cancer. 2013;13:2. CrossRef. Medline 23282173 10.1186/1471-2407-13-2 PMC3559275

[12] Mitiku I, Tefera F. Knowledge about cervical cancer and associated factors among 15-49 year old women in Dessie Town, Northeast Ethiopia. PLoS One. 2016;11(9):e0163136. 10.1371/journal.pone.0163136. Medline 27690311 PMC5045174

[13] Wong LP, Wong YL, Low WY, Khoo EM, Shuib R. Knowledge and awareness of cervical cancer and screening among Malaysian women who have never had a Pap smear: a qualitative study. Singapore Med J. 2009;50(1):49–53. Medline 19224084

[14] Mukama T, Ndejjo R, Musabyimana A, Halage AA, Musoke D. Women's knowledge and attitudes towards cervical cancer prevention: a cross sectional study in Eastern Uganda. BMC Womens Health. 2017;17:9. 10.1186/s12905-017-0365-3. Medline 28137260 PMC5282746

[15] Raychaudhuri S, Mandal S. Current status of knowledge, attitude and practice (KAP) and screening for cervical cancer in countries at different levels of development. Asian Pac J Cancer Prev. 2012;13(9):4221–4227. 10.7314/apjcp.2012.13.9.4221. Medline 23167318

[16] Gan D EH, Dahlui M. Cervical screening uptake and its predictors among rural women in malaysia. Singapore Med J. 2013;54(3):163–168. 10.11622/smedj.2013047. Medline 23546031

[17] Balogun MR, Odukoya OO, Oyediran MA, Ujomu PI. Cervical cancer awareness and preventive practices: a challenge for female urban slum dwellers in Lagos, Nigeria. Afr J Reprod Health. 2012;16(1):75–82. Medline 22783671

[18] Armstrong CE, Martí-nez-Álvarez M, Singh NS, et al. Subnational variation for care at birth in Tanzania: is this explained by place, people, money or drugs? BMC Public Health. 2016;16(suppl 2):795. 10.1186/s12889-016-3404-3. Medline 27634353 PMC5025821

[19] Lyimo FS, Beran TN. Demographic, knowledge, attitudinal, and accessibility factors associated with uptake of cervical cancer screening among women in a rural district of Tanzania: three public policy implications. BMC Public Health. 2012;12:22. 10.1186/1471-2458-12-22. Medline 22233530 PMC3299640

[20] Ebu NI, Mupepi SC. Knowledge, practice, and barriers toward cervical cancer screening in Elmina, Southern Ghana. Int J Womens Health. 2014;7:31–39. 10.2147/IJWH.S71797. Medline 25565902 PMC4284003

[21] Kivistik A, Lang K, Baili P, Anttila A, Veerus P. Women's knowledge about cervical cancer risk factors, screening, and reasons for non-participation in cervical cancer screening programme in Estonia. BMC Womens Health. 2011;11:43. 10.1186/1472-6874-11-43. Medline 21951661 PMC3192747

[22] Lofters AK, Moineddin R, Hwang SW, Glazier RH. Predictors of low cervical cancer screening among immigrant women in Ontario, Canada. BMC Womens Health. 2011;11:20. 10.1186/1472-6874-11-20. Medline 21619609 PMC3121675

[23] Peirson L, Fitzpatrick-Lewis D, Ciliska D, Warren R. Screening for cervical cancer: a systematic review and meta-analysis. Syst Rev. 2013;2:35. 10.1186/2046-4053-2-35. Medline 23706117 PMC3681632

[24] Moshi FV, Vandervort EB, Kibusi SM. Cervical cancer awareness among women in Tanzania: an analysis of data from the 2011-12 Tanzania HIV and Malaria Indicators Survey. Int J Chronic Dis. 2018;2018:2458232. 10.1155/2018/2458232. Medline 29854721 PMC5954879

[25] Shiferaw N, Brooks MI, Salvador-Davila G, et al. Knowledge and awareness of cervical cancer among HIV-infected women in Ethiopia. Obstet Gynecol Int. 2016;2016:1274734. 10.1155/2016/1274734 27867397 PMC5102747

[26] Ali-Risasi C, Mulumba P, Verdonck K, Van den Broeck D, Praet M. Knowledge, attitude and practice about cancer of the uterine cervix among women living in Kinshasa, the Democratic Republic of Congo. BMC Womens Health. 2014;14:30. 10.1186/1472-6874-14-30. Medline 24548698 PMC3937079

[27] Kasa AS, Tesfaye TD, Temesgen WA. Knowledge, attitude and practice towards cervical cancer among women in Finote Selam City Administration, West Gojjam Zone, Amhara Region, North West Ethiopia, 2017. Afr Health Sci. 2018;18(3):623–636. 10.4314/ahs.v18i3.20. Medline 30602995 PMC6307012

[28] Nelson SB, Viswanathan N, Jenifer NA, Priyanka B. A cross-sectional study on cervical cancer and its prevention among women of age group 25–50 years in a rural area of South Tamil Nadu, India. Int J Community Med Public Health. 2018;5(6):2536–2541. 10.18203/2394-6040.ijcmph20182191

[29] Begum R, Shuayb MD. Knowledge about carcinoma cervix among the females of reproductive age group in selected urban communities in Bangladesh. Adv Cancer Prev. 2016;1(3):1000114. 10.4172/2472-0429.1000114

[30] Cunningham MS, Skrastins E, Fitzpatrick R, et al. Cervical cancer screening and HPV vaccine acceptability among rural and urban women in Kilimanjaro Region, Tanzania. BMJ Open. 2015;5(3):e005828. 10.1136/bmjopen-2014-005828. Medline PMC4360576 25757944

[31] Mukona DM, Ndaimani A, Maxwell M. Knowledge of cervical cancer and uptake of cervical cancer screening in women aged 18–45 years at a central hospital in Harare, Zimbabwe. Nova J Med Biol Sci. 2018;6(1). Crossref

[32] Kahesa C, Kjaer S, Mwaiselage J, et al. Determinants of acceptance of cervical cancer screening in Dar es Salaam, Tanzania. BMC Public Health. 2012;12:1093. 10.1186/1471-2458-12-1093. Medline 23253445 PMC3551792

[33] Ndejjo R, Mukama T, Musabyimana A, Musoke D. Uptake of cervical cancer screening and associated factors among women in rural Uganda: a cross sectional study. PLoS One. 2016;11(2):e0149696. 10.1371/journal.pone.0149696. Medline 26894270 PMC4760951

